# Determination of fitness traits of *Orius strigicollis* Poppius (Hemiptera: Anthocoridae) on *Pectinophora gossypiella* (Lepidoptera: Gelechiidae) using two-sex life table analysis

**DOI:** 10.7717/peerj.9594

**Published:** 2020-08-17

**Authors:** Shahzaib Ali, Qian Zhu, Waqar Jaleel, Shakeel Ur Rehman, Muhammad Asim Rasheed, Muhammad Musa Khan, Yasir Islam, Muhammad Hafeez, Xingmiao Zhou

**Affiliations:** 1 Hubei Insect Resources Utilization and Sustainable Pest Management Key Laboratory, College of Plant Science and Technology, Huazhong Agricultural University, Wuhan, China; 2 Guangdong Provincial Key Laboratory of High Technology for Plant Protection, Plant Protection Research Institute, Guangdong Academy of Agricultural Sciences, Guangzhou, China; 3 Key Laboratory of Bio-Pesticide Innovation and Application, Engineering Research Centre of Biological Control, South China Agricultural University, Guangzhou, China; 4 State Key Laboratory Breeding Base for Zhejiang Sustainable Pest and Disease Control, Institute of Plant Protection and Microbiology, Zhejiang Academy of Agricultural Sciences, Hangzhou, Zhejiang, China

**Keywords:** Age-stage, Feeding potential, Fitness, *Orius strigicollis*, *Pectinophora gossypiella*, Population parameters, Two-sex life table, Temperature

## Abstract

**Background:**

Pink bollworm (*Pectinophora gossypiella*) is a destructive insect pest of cotton crops in China and globally, which is actively predated on by *Orius strigicollis*. Studies on the fitness or survival of *O. strigicollis* fed on *P. gossypiella* at different temperatures have not been reported. The fitness of *O. strigicollis* may be well explained using two-sex life table parameters. Thus, the present study provides important insights for the effective biocontrol of *P. gossypiella*.

**Methodology:**

Considering the importance of fitness parameters and biocontrol, the present study explores the feeding potential and age-stage, two-sex life table traits of *O. strigicollis* on *P. gossypiella* eggs at different temperatures (24, 28 and 32 °C) in the laboratory.

**Results:**

The intrinsic rate of increase (*r*) was higher at 28 °C (0.14 d^−1^) than at 24 °C (0.0052 d^−1^) and 32 °C (0.12 d^−1^). Similarly, the net reproductive rate (*R*_0_) was higher at 28 °C (17.63 offspring) than at 24 °C (1.13 offspring) and 32 °C (10.23 offspring). This concluded that the maximum feeding potential and growth capacity of *O. strigicollis* could be attained at 28 °C when fed on *P. gossypiella* eggs. *O. strigicollis* adults preferred to feed on *P. gossypiella* eggs compared with first instar larvae. Based on these results, the present study suggests that *O. strigicollis* represents a promising biological control agent against *P. gossypiella* eggs in cotton fields.

## Introduction

The biology of predators and parasitoids is greatly influenced by environmental factors (Jaleel et al., 2019; Ali et al., 2020). Temperature is one of the most important abiotic environmental factors (Saleh & Sengonca, 2003; Jaleel et al., 2020). The development time of insect pests is greatly affected by variable temperature (Barbosa et al., 2019). Understanding the fitness of predators and parasitoids under different environmental conditions is important for the establishment of successful biocontrol programs (Chen et al., 2018b; Kutcherov, Lopatina & Yermakov, 2019).

Cotton is the most dynamic cash crop in 111 countries and is known as the “King of fibres” or “White gold” (Parmar & Patel, 2016). China is the largest producer of cotton crop worldwide. The average lint yield of cotton was 1,438 kg ha^−1^ from 5.3 million ha of cultivated area (Yosoff et al., 2015). Over the last 20 years, the cotton crop has been continuously damaged by several insect pests. Among cotton pests, *Pectinophora gossypiella* (Saunders) (Lepidoptera: Gelechiidae) is a serious pest (Ahmad et al., 2011). Currently, *P. gossypiella* is a monophagous pest that is, damaging the cotton boll in China (Wan et al., 2012; Edpuganti, 2018). Control of *P. gossypiella* is difficult through the application of insecticides owing to the concealed mode of feeding (Naranjo, Butler & Henneberry, 2002). Worldwide, *P. gossypiella* has become the most devastative insect pest of cotton and has been identified to cause 2.8–61.9% loss in seed cotton yield, 2.1–47.1% loss in oil content, and 10.7–59.2% loss in the normal opening of bolls (Patil, 2003). The best and safest option for reducing the population of *P. gossypiella* is biocontrol by use of, for example, predators.

*Orius* spp. are the best predators against lepidopteran pests, because they exhibit a higher searching efficiency for their host, and are fast moving and active (Minks & Harrewijn, 1988). *Orius* spp. have also been demonstrated to be the best predators against aphids (Hemiptera: Aphididae), whiteflies (Hemiptera: Aleyrodidae), mites (Arachnida: Acaridae), young lepidopterous larvae and small arthropod eggs (Bonte et al., 2015). Among *Orius* spp., *Orius strigicollis* Poppius (Heteroptera: Anthocoridae) which was previously known as *Orius similis* Zheng (Heteroptera: Anthocoridae) (junior synonym of *O. strigicollis*) (Yasunaga, 1997; Jung, Yamada & Lee, 2013), also known as the predatory flower bug, originated in southern China and is a more effective predator in agroecosystems (Zhou et al., 2006). Adults and nymphs of *O. strigicollis* feed on an extensive range of soft bodied insect pests, such as *Aphis gossypii*, *Frankliniella formosae* (Moulton) and *Tetranychus cinnabarinus* (Boisduval) (Zhang et al., 2012), and also feed on lepidopteran pest eggs or hatched larvae (Bonte & De Clercq, 2011; Ali et al., 2020). *O. strigicollis* have been observed in large quantities in cotton fields (Zhou & Lei, 2002; Ahmadi, Sengonca & Blaeser, 2007).

The biological characteristics of some *Orius* species have been reported at different temperatures and when feeding on different prey species. The developmental duration of *O. insidiosus* (Kim, 1997, 1999), *O. albidipennis* (Reuter), and *O. laevigatus* (Fieber) have been reported (Kiman & Yeargan, 1985). However, fitness studies on *O. strigicollis* are limited. Zhou & Lei (2002) and Zhang et al. (1994) studied the fitness parameters of *O. similis* when fed on *A. gossypii* and *P. gossypiella*, respectively, but further research is needed to fully understand these parameters. Ahmadi, Sengonca & Blaeser (2007) studied the effect of two temperatures (18 °C and 30 °C) on the biology of *O. similis* fed on two aphid species. Ali et al. (2020) studied the fitness of *O. strigicollis* on different densities of *P. gossypiella* eggs. To the best of our knowledge, detailed information about the feeding potential and fitness of *O. strigicollis* using age-stage, two-sex life table on *P. gossypiella* eggs at different temperatures is lacking in the literature.

In integrated pest management (IPM), biocontrol is the preferred option against *P. gossypiella* because it is environmentally safe and nontoxic (Dhawan, Kumar & Shera, 2011; Pandey et al., 2019). In IPM programs, it is necessary to understand both the basic and detailed information of *O. strigicollis*, which can be derived from life table modeling (Jaleel, Lu & He, 2018; Ali et al., 2020). However, traditional life table models only provide information for female adults, whereas models for both male and female adults are necessary to understand population dynamics (Huang & Chi, 2013; Chen et al., 2017). The establishment of biological control agents for *P. gossypiella* in the field is an important task for researchers. In the present study, feeding potential and fitness parameters of *O. strigicollis* were studied when they were fed on *P. gossypiella* eggs at three different constant temperatures in a laboratory using the age-stage, two-sex life table traits. The present study provides basal information for developing effective biocontrol agents against *P. gossypiella*.

## Materials and Methods

### Insect rearing

#### Rearing of *O. strigicollis*

Adults of *O. strigicollis* were collected from different fields of cotton flowers at Huazhong Agricultural University, Wuhan, Hubei Province, P.R. China. Culture was maintained on *P. gossypiella* eggs in the laboratory according to a previously described method (Zhou et al., 2006). The population of *O. strigicollis* was maintained on the *P. gossypiella* eggs at three temperatures (24, 28 and 32 °C) to acclimatize for at least two generations before the experiments (Jaleel et al., 2020). The reason for the selection of three temperatures (24, 28 and 32 °C) was that many *Orius* spp. including *O. strigicollis* showed better performance/predation against different insect pests in different laboratory studies (Zhang et al., 2012; Amer, Fu & Niu, 2018; Ali et al., 2020), and mainly these temperatures are favorable for many lepidopterous insect pests damaging stages including *P. gossypiella* (Ali et al., 2016; Shrinivas et al., 2019; Umer, Atif & Ahmad, 2019). Small tender stems (3–4) of *Vitex negundo* L. (Lamiaceae: Verbenaceae) were provided as oviposition substrate in plastic containers (100 mm wide base and 124 mm deep) under laboratory conditions. Stems were wrapped in wet cotton at the end and refreshed after two days. Stems containing *O. strigicollis* eggs were harvested daily and kept in separate containers until adult emergence.

### Collection of *P. gossypiella*

Pupae and adults of the pink bollworm *P. gossypiella* (strain resistant AZP-R) were supplied by the Institute of Plant Protection and Soil Fertility, Hubei Academy of Agricultural Sciences, Wuhan, China, and were placed in cages (50 × 50 × 50 cm) and provided with 10% honey solution for egg laying as a diet for adults. Each cage was covered with white gauze and filter paper for oviposition. Egg masses were harvested or collected daily on the filter paper during the total oviposition period following previously described methods (Wan et al., 2012), and fresh eggs were provided as a food source for the flower bugs. All experiments were conducted at 75 ± 5% RH and a 16:8 (L:D) photoperiod in climatic controlled chambers (HP350GS, Ruihua Instrument & Equipment Co., Ltd., Wuhan, P.R. China) equipped with fluorescent lighting controlled by an automatic timer.

### Feeding potential of *O. strigicollis*

The feeding potential of *O. strigicollis* predatory stages, that is, third, fourth and fifth nymphal instars and adult stages (male and female), was recorded on *P. gossypiella* eggs and first instar larvae on the basis of choice experiments. The feeding efficiency of all predatory stages with *P. gossypiella* eggs after 24 h, and for first instar larvae after 12 and 24 h was determined in three temperature treatments (24, 28 and 32 °C), in plastic Petri dishes (9 cm diameter and 2 cm depth), lined with filter paper. There were 30 replicates for each predatory stage with 20 prey (eggs and larvae). Owing to zero percent mortality of fresh and healthy *P. gossypiella* eggs at three different temperatures in our main experiments, we did not take them into account while analyzing the data and there was no control treatment for *O. strigicollis* potential on *P. gossypiella* eggs. Although there were 30 replicates of the control treatment without predators at three temperatures after 12 and 24 h for *O. strigicollis* potential on *P. gossypiella* first instar larvae. The control treatment was considered as a prediction of *P. gossypiella* first instar larvae mortality with or without predators to check the actual feeding potential of *O. strigicollis* predatory stages on *P. gossypiella* first instar larvae by following previously described methodology (Calixto et al., 2013).

### Two-sex life table traits

The *O. strigicollis* eggs in bulk quantity (100) at each temperature were selected and the development time was checked from egg hatching to second instar nymph in groups (≤10 in each box) when *P. gossypiella* eggs were provided as diet and cotton balls drenched with water to maintain the moisture level and source of water for immature nymphs. Beginning with the third instar nymphal stage (*N*_3_), individual bugs were isolated in plastic Petri dishes lined with filter paper to avoid cannibalism (Tuan et al., 2015), and each individual was considered as a replicate at each temperature until death to check the biological parameters of *O. strigicollis*. Each nymph was fed until adult emergence, and the stadial duration of each stage was noted after every 24 h. Fresh *P. gossypiella* eggs were provided daily, and after every 24 h, consumed or damaged eggs were counted under a stereomicroscope (1309 LED 40X Binocular Stereo Microscope; Jiangsu Victor Instrument Meter Co., Ltd., Taizhou, JS, China), and magnifying lens, and dead individuals of *O. strigicollis* were counted to check the mortality of *O. strigicollis*.

Moulting or shredded skin of *O. strigicollis* nymphs in the round plastic Petri dishes were checked daily to assess the next nymphal stage (Ahmadi, Sengonca & Blaeser, 2007). From last instar nymph (fifth) to adult emergence was checked and male/female sex was identified under a stereomicroscope as documented by (Ali et al., 2020), and kept separately and starved for 24 h for the reproductive study.

For the adult longevity and egg laying capacity of *O. strigicollis*, both females and males were shifted to new cylindrical glassy vials (2.6 cm diameter and 15 cm length) enclosed with a fine mesh nylon screen. The pairs were noted to ensure that mating occurred, and the females that continued copulation for >1.5 minutes were considered to have been mated (Butler & O’Neil, 2006). The pairs at each temperature were placed in Petri dishes lined with filter paper, and *P. gossypiella* eggs were provided as food source. The small fragile stem of *Vitex negundo* L. was provided as a substrate for female *O. strigicollis* oviposition (Zhou et al., 2006) at each temperature (24, 28 and 32 °C). The stem was covered with moistened cotton as described above.

The stems were investigated daily under a stereomicroscope to count the number of eggs laid by *O. strigicollis*. Consumed or damaged eggs of *P. gossypiella* were counted and replaced with fresh eggs daily. The following biological parameters were recorded at each temperature: adult pre-oviposition period (APOP = the time period between the female adult emergence to its first egg laying), total pre-oviposition period (TPOP = the time interval between birth to the start of egg laying), oviposition days (taking only those days (time units) with fecundity >0 into account), and daily fecundity following previously described methods (Huang & Chi, 2013; Chen et al., 2017). The stems with eggs laid by *O. strigicollis* were kept in plastic containers at each temperature, 70% ± 5% (RH%) and L16:D8 photoperiod. The number of *O. strigicollis* hatched eggs were recorded daily under a stereomicroscope. The percentage of egg hatchability and mating pair success was recorded per pair. All *O. strigicollis* bugs (female) were kept and observed until death following the methodology of Zhang et al. (2012).

### Prey preference

The predatory stages of *O. strigicollis* third, fourth, fifth instars and males and females were exposed to 10 prey/host stages of both *P. gossypiella* eggs and first instar larvae in the same plastic Petri dishes at three different temperatures (24, 28 and 32 °C). There were 10 replicates for each predatory stage at each temperature. Each replicate was provided with moistened cotton placed inside the Petri dish to maintain the moisture and a water source for all predatory stages in the above-mentioned controlled chamber. The preferred or consumed host/prey were examined after 12-and 24-h intervals at each temperature under a stereomicroscope.

### Statistical analysis

In this study, the data of feeding potentials of *O. strigicollis* on *P. gossypiella* eggs and larvae at three different temperatures were statistically analyzed using one-way analysis of variance (ANOVA), and their mean values were compared using least significant difference (LSD) test at the *p* = 0.05 level of significance. The correlation between different temperatures and feeding potential of predatory stages was determined by linear regression analysis (Saeed et al., 2016). All statistical analyses were carried out using statistics 8.1 software (Analytical Software, Tallahassee, FL, USA). Different biological parameters (each stage developmental period, survival rate, adult longevity, age-specific fecundity, APOP and TPOP) were statistically evaluated using age-stage two-sex life table theory (Chi & Liu, 1985; Chi, 1988) and the TWOSEX-MS Chart computer program (Chi, 2015; Hafeez et al., 2019). Means and standard errors (SE) of all biological and population life table parameters were determined using 200,000 bootstrap replicates to obtain stable SE estimates (Huang & Chi, 2012; Akca et al., 2015). Bootstrap and paired bootstrap tests were figured in TWOSEX-MS Chart, and all results of the treatments were associated using the paired bootstrap test based on the confidence interval of difference (Efron & Tibshirani, 1993; Akköprü et al., 2015). The age-stage-specific survival rate (*s_xj_*), age-stage-specific fecundity (*f_xj_*), age-specific survival rate (*l_x_*), age-specific fecundity (*m_x_*), age-stage life expectancy (*e_xj_*), age-stage reproductive value (*v_xj_*), and life table parameters (*r*, intrinsic rate of increase; λ, finite rate of increase; *R*_0_, net reproductive rate; and *T*, the mean generation time), were designed in sequence according to previously described methods (Chi & Liu, 1985; Tuan et al., 2015). Sigma Plot 12.0 was used to construct the curves for all population or life table parameters, including *s_xj_*, *f_xj_*, *m_x_*, *v_xj_* and *e_xj_*.

The age-specific survival rate (*l_x_*) and age-specific fecundity (*m_x_*) were calculated as follows:

(1) $${l_x} = \mathop \sum \limits_{j = 1}^k {{\rm s}_{xj}}$$

(2) $${m_x} = \displaystyle{{\mathop \sum \nolimits_{j = 1}^k {s_{xj\; }}{f_{xj}}} \over {\mathop \sum \nolimits_{j = 1}^k {s_{xj}}}}$$

where *s_xj_* is the age-stage specific survival rate, that is, the possibility that an individual (newly hatched) will live or exist to age *x* and stage *j*. The intrinsic rate of increase (*r*) was then predicted iteratively following the Euler–Lotka equation with age indexed from 0 (Goodman, 1982) as follows:

(3) $$\mathop \sum \limits_{x = 0}^\infty {{\rm e}^{ - r\left( {x + 1} \right)}}{l_{x\; }}{m_x} = 1$$

The net reproductive rate *R*_0_ was calculated as follows:

(4) $${R_0} = \mathop \sum \limits_{x = 0}^\infty {l_{x\; }}{m_x}$$

The net reproductive rate (*R*_0_) and mean female fecundity (*F*) relationship was calculated as follows:

(5) $${R_0} = \; \; F\; \displaystyle{{{N_f}} \over N}$$

where *N* indicates the total number of individuals used for the life table study and *N*_*i*_ represents the number of female adults (Chi, 1988). The gross reproduction rate was defined as follows:

(6) $$GRR = \mathop \sum \limits_{x = 0}^\infty {m_x}$$

The finite rate (λ) was recorded as follows:

(7) $${\rm{\lambda}} = {e^r}$$

The mean generation time (*T*) represents the time span that a population needs to increase to *R*_0_-fold of its size, that is, ${e^{rT}} = {R_0}$ or ${\lambda ^T} = {R_0}$ at the stable age-stage distribution. The value of *T* was calculated as follows:

(8) $$T = \displaystyle{{{\rm ln}{R_0}} \over r}$$

Age-stage life expectancy (*e_xj_*) is defined as the length of the duration or time that an individual or insect of *x* and *j* is predicted to live, calculated by the method of (Chi & Su, 2006) as follows:

(9) $${e_{xj}} = \; \mathop \sum \limits_{i = x}^\infty \mathop \sum \limits_{y = j}^k s^{\prime}_{iy}$$

Where $s^{\prime}_{iy}$ is defined as the probability that individuals of *x* and *j* will survive to age *i* and stage *y*, and is found by assuming $s^{\prime}_{iy}$ =1 (Tuan, Lee & Chi, 2014).

The age-stage reproductive value (*v_xj_*) was defined as the contribution of individuals of age *x* and stage *j* to the future population (Yang et al., 2015). In the age-stage, two-sex life table, it was calculated as follows (Tuan, Lee & Chi, 2014):

(10) $${v_{xj}} = \; \displaystyle{{{e^{ - r\left( {x + 1} \right)}}} \over {{S_{xj}}}}\; \mathop \sum \limits_{i = x}^\infty {e^{ - r\; \left( {x + 1} \right)}}\mathop \sum \limits_{y = j}^k s^{\prime}_{iy}\,{f_{iy}}$$

Prey preference between two prey types for example, *P. gossypiella* eggs and first instar larvae was determined using a paired *t*-test.

## Results

### Feeding potential of *O. strigicollis*

**Figure 1:**
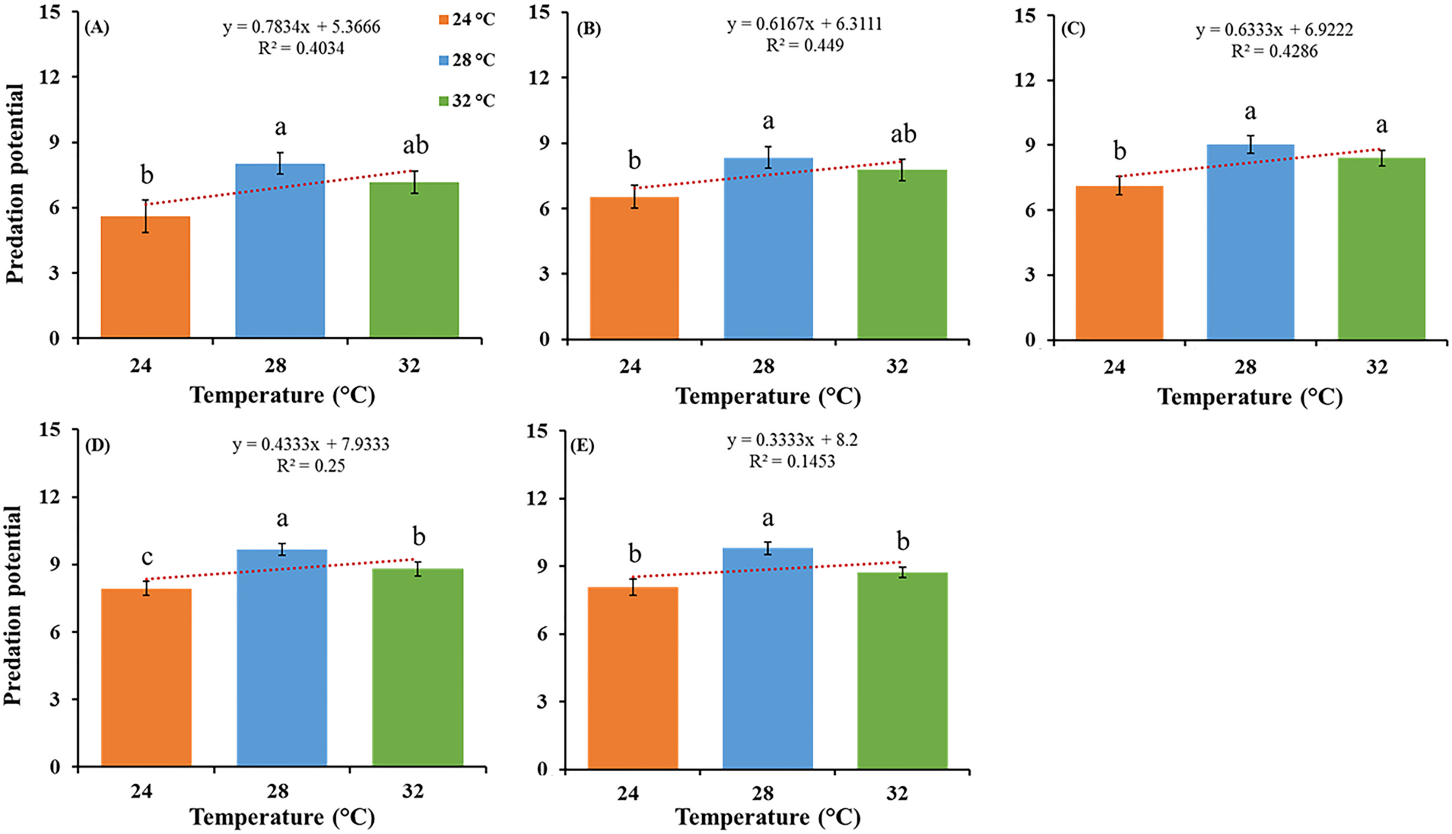
Feeding potential of the predatory stages of the *O. strigicollis* at three different temperatures (24 °C, 28 °C and 32 °C) on *P. gossypiella* eggs after 24 h. Means marked with different letters are significantly different between three treatments (One-way ANOVA, LSD test, *p* < 0.01, *n* = 30). (A) Third instar, (B) fourth instar, (C) fifth instar, (D) male and (E) female.

The feeding potential of *O. strigicollis* predatory stages, that is, third, fourth, fifth nymphal instars and adult stages (male and female) and correlations between different temperatures, when fed on *P. gossypiella* eggs (Fig. 1), and first instar larvae were recorded (Table 1). Among all predatory stages on *P. gossypiella* eggs, females showed significantly more feeding capability at 28 °C (*F*_2,87_ = 8.89; *P* < 0.001) than those in the two other treatments at 24 °C and 32 °C after 24 h. Similarly, males showed significantly more feeding capability at 28 °C (*F*_2,87_ = 8.33; *P* < 0.001) than those in the other two treatments at 24 °C and 32 °C after 24 h (Fig. 1).

**Table 1:** Feeding potential of *O. strigicollis* on *P. gossypiella* first instar larvae at three different temperatures.

Stages	Temperature (12 h interval)			Regression	ANOVA			Temperature (24 h interval)			Regression	ANOVA		
	24 °C	28 °C	32 °C	*R*^2^, y	*df*	*F*	*P*	24 °C	28 °C	32 °C	*R*^2^, y	*df*	*F*	*P*
Third instar nymph	3.13 ± 0.24 c	6.63 ± 0.31 a	5.03 ± 0.31 b	0.294, 0.95x + 3.03	2, 87	36.90	0.0000	4.87 ± 0.35 c	7.80 ± 0.26 a	6.90 ± 0.30 b	0.458, 1.02x + 4.49	2, 87	24.10	0.0000
Fourth instar nymph	4.33 ± 0.30 c	7.07 ± 0.26 a	5.57 ± 0.34 b	0.203, 0.62x + 4.42	2, 87	20.40	0.0000	5.87 ± 0.35 c	8.37 ± 0.30 a	7.30 ± 0.37 b	0.326, 0.72x + 5.74	2, 87	13.60	0.0000
Fifth instar nymph	5.27 ± 0.40 b	6.90 ± 0.28 a	6.47 ± 0.39 a	0.503, 0.60x + 5.01	2, 87	5.53	0.0055	7.13 ± 0.33 b	8.50 ± 0.25 a	9.30 ± 0.35 a	0.978, 1.08x + 6.14	2, 87	12.00	0.0000
Male	7.43 ± 0.31 b	10.43 ± 0.23 a	9.77 ± 0.18 a	0.549, 1.17x + 6.88	2, 87	41.00	0.0000	9.70 ± 0.28 b	11.17 ± 0.15 a	11.27 ± 0.16 a	0.798, 0.78x + 9.14	2, 87	18.20	0.0000
Female	8.27 ± 0.30 b	10.07 ± 0.28 a	9.80 ± 0.27 a	0.623, 0.77x + 7.84	2, 87	11.80	0.0000	9.90 ± 0.30 b	11.20 ± 0.23 a	11.57 ± 0.13 a	0.905, 0.83x + 9.22	2, 87	14.40	0.0000
Control	2.90 ± 0.31 b	3.43 ± 0.31 b	4.90 ± 0.31 a	0.932, 0.00x + 1.74	2, 87	11.30	0.0000	4.20 ± 0.25 b	4.70 ± 0.30 b	5.77 ± 0.31 a	0.958, 0.78x + 3.32	2, 87	7.79	0.0008

**Note:**

Means marked with different letters are significantly different between three treatments using LSD test, *p* < 0.05 and *n* = 30.

Similarly, all predatory stages fed with *P. gossypiella* larvae showed different feeding capability with varying temperature after 12-and 24-h intervals. Females showed significantly more feeding at 28 °C (*F*_2,87_ = 11.80; *P* < 0.0001) than those in the other two treatments at 24 °C and 32 °C after 12 h, and males showed significantly more feeding at 28 °C (*F*_2,87_ = 41.00; *P* < 0.0001) than those in the other two treatments at 24 °C and 32 °C after 12 h (Table 1). Meanwhile, females showed significantly more feeding at 32 °C (*F*_2,87_ = 14.40; *P* < 0.0001) than those in the other two treatments at 24 °C and 28 °C after 24 h, and males showed significantly more feeding at 32 °C (*F*_2,87_ = 18.20; *P* < 0.0001) than those in the other two treatments at 24 °C and 28 °C after 24 h (Table 1). There were no differences recorded in male and female average predation on *P. gossypiella* first instar larvae after 12-and 24-h intervals. For the control treatment (*F*_2,87_ = 11.30; *P* < 0.0001) after 12 h, the mortality of *P. gossypiella* first instar larvae was significantly higher at 32 °C than that in the other two treatments at 24 °C and 28 °C after 12 h. Similarly, for control treatment (*F*_2,87_ = 7.79; *P* < 0.001) after 24 h, the mortality of *P. gossypiella* first instar larvae was significantly higher at 32 °C than that in the other two treatments at 24 °C and 28 °C after 24 h. Overall, predatory stages feeding after 12-and 24-h were significantly different as compared to control treatment (Table 1).

### Biological parameters of *O. strigicollis*

#### Developmental period

The development time (days) from egg to pre-adult stages differed at each temperature (24, 28 and 32 °C) when fed on the *P. gossypiella* eggs (Table 2). The minimum egg development period was observed at 32 °C (2.87 ± 0.04 d) compared with those at the other two temperatures: 24 °C (4.27 ± 0.06 d) and 28 °C (3.23 ± 0.06 d). There were differences recorded in mean development time of third instars at 24 °C (2.05 ± 0.12 d) compared with those at the other two temperatures: 28 °C (2.31 ± 0.20 d) and 32 °C (2.83 ± 0.08 d), and fourth instars at 24 °C (2.71 ± 0.17 d) compared with those at the other two temperatures: 28 °C (1.93 ± 0.11 d) and 32 °C (2.82 ± 0.11 d). There were no differences recorded in mean survival time of adult males whereas, the differences were recorded in mean survival time of adult females at the three tested temperatures (Table 2).

**Table 2:** Effect of three different temperatures on the development traits of *O. strigicollis* on *P. gossypiella* eggs.

Stages	Temperature			ANOVA		
	24 °C	28 °C	32 °C	*df*	*F*	*P*
Egg duration (d)	4.27 ± 0.06 a	3.23 ± 0.06 b	2.87 ± 0.04 c	2,271	305	0.0000
First instar nymph (d)	3.17 ± 0.05 a	3.00 ± 0.00 b	2.17 ± 0.05 c	2,244	221	0.0000
Secone instar nymph (d)	3.00 ± 0.00 a	2.93 ± 0.03 b	2.00 ± 0.00 c	2,214	840	0.0000
Third instar nymph (d)	2.05 ± 0.12 b	2.31 ± 0.20 b	2.83 ± 0.08 a	2,172	16.0	0.0001
Fourth instar nymph (d)	2.71 ± 0.17 a	1.93 ± 0.11 b	2.82 ± 0.11 a	2,148	26.2	0.0000
Fifth instar nymph (d)	3.88 ± 0.15 a	3.93 ± 0.15 a	2.47 ± 0.09 b	2,127	78.4	0.0000
Male survival (d)	7.50 ± 0.29 a	6.67 ± 1.25 a	6.12 ± 1.11 a	2,51	0.17	0.8417
Female survival (d)	5.33 ± 0.86 b	8.88 ± 2.13 a	7.89 ± 1.17 ab	2,73	2.47	0.0916
Total longevity of male adult (d)	26.00 ± 0.58 a	23.67 ± 1.08 ab	21.25 ± 1.18 b	2,51	3.60	0.0345
Total longevity of female adult (d)	23.83 ± 0.86 a	26.12 ± 2.10 a	23.33 ± 1.23 a	2,73	1.02	0.3673
TPOP/TPRP^a^ (d)	21.50 ± 0.42 a	17.50 ± 0.50 b	15.56 ± 0.30 c	2,65	47.1	0.0000
APOP/APRP^b^ (d)	3.00 ± 0.46 a	0.25 ± 0.11 b	0.11 ± 0.08 b	2,65	99.2	0.0000
Oviposition (d)	3.75 ± 0.31 b	7.88 ± 1.92 a	5.89 ± 0.78 ab	2,65	3.17	0.0484
Post-Oviposition (d)	0.33 ± 0.29 a	0.50 ± 0.25 a	0.67 ± 0.23 a	2,65	1.03	0.3612
Fecundity (eggs /female)	5.67 ± 1.38 c	66.12 ± 9.38 a	34.11 ± 6.8 b	2,65	20.6	0.0000
MPS^c^ (%)	66.67 ± 10.11 b	92.59 ± 6.74 a	86.21 ± 6.50 ab	2,65	2.30	0.0424
Hatchability (%)	39.24 ± 2.47 b	54.37 ± 1.65 a	31.07 ± 1.59 c	2,65	52.5	0.0000

**Notes:**

a TPOP, total pre-oviposition period of female; TPRP, total pre-reproduction period of female.

b APOP, adult pre-oviposition period of female; APRP, adult pre-reproduction period of female.

c MPS, mating pair success.

The standard errors of the mean (SEM) values were estimated by using 200,000 bootstrap replicates. df, degree of freedom (treatment and error). Means marked with different letters are significantly different between three treatments using the paired bootstrap test at the 5% significant level.

### Longevity, oviposition and fecundity of adults

The mean total longevity of male adults considerably declined with increasing temperature (26.00 d to 21.25 d), whereas the mean total longevity of female adults was the highest at 28 °C (26.12 d) as compared with the other temperatures at 24 °C (23.83 d) and 32 °C (23.33 d) (Table 2). However, there were no differences recorded in the total longevity of female adults among the three treatment temperatures. The TPOP of female adults differed at 24 °C (21.50 ± 0.42 d), 28 °C (17.50 ± 0.50 d), and 32 °C (15.56 ± 0.30 d) when fed on the *P. gossypiella* eggs. Similarly, the APOP of female adults differed at 24 °C (3.00 ± 0.46 d), 28 °C (0.25 ± 0.11 d) and 32 °C (0.11 ± 0.08 d) when fed on the *P. gossypiella* eggs. The oviposition period of female adults differed at 28 °C (7.88 ± 1.92 d) as compared with the other temperatures at 24 °C (3.75 ± 0.31 d) and 32 °C (5.89 ± 0.78 d). There were differences recorded in fecundity (total number of eggs per female), and the highest fecundity was recorded at 28 °C (66.12 eggs/female) compared with the other two temperatures. The mating pair success (the percentage of pairs of adults that successfully laid eggs or were eligible to continue in the next generation) of *O. strigicollis* at three different temperatures was different, and the highest percentage of MPS was recorded at 28 °C (92.59%). Similarly, the hatching percentage of eggs laid by *O. strigicollis* females at three different temperatures was different, and the highest percentage of hatched eggs was recorded at 28 °C (54.37%) (Table 2).

### Population parameters

The effects of different temperatures on population or fitness parameters of *O. strigicollis* fed on the *P. gossypiella* eggs were calculated by using bootstrap technique with 200,000 resamplings (Table 3). The intrinsic rate of increase (*r*) and finite rate of increase (λ) of *O. strigicollis* fed on the *P. gossypiella* eggs differed and were higher at 28 °C (0.14 ± 0.01 d^−1^ and 1.15 ± 0.02 d^−1^) compared with those at 24 °C (0.0052 ± 0.002 d^−1^ and 1.00 ± 0.02 d^−1^) and 32 °C (0.12 ± 0.02 d^−1^ and 1.13 ± 0.02 d^−1^). In addition, the net reproductive rate (*R*_0_) was the highest and different at 28 °C (17.63 offspring) compared with those at the other two temperatures: 24 °C (1.13 offspring) and 32 °C (10.23 offspring). Conversely, the mean generation time (*T*) was the highest and different at 24 °C (23.79 days) compared with those at the other two temperatures: 28 °C (20.89 days) and 32 °C (19.60 days). Gross reproductive rate (*GRR*) was different and the highest at 28 °C (90.32) compared with those at the other two temperatures: 24 °C (6.18) and 32 °C (30.76) (Table 3).

**Table 3:** Effect of three different temperatures on the population parameters of *O. strigicollis* on *P. gossypiella* eggs.

Population parameters	Temperature		
	24 °C	28 °C	32 °C
Intrinsic rate of increase (*r*) (d^−1^)	0.0052 ± 0.002 b	0.14 ± 0.01 a	0.12 ± 0.02 a
Finite rate of increase (*λ*) (d^−1^)	1.00 ± 0.02 b	1.15 ± 0.02 a	1.13 ± 0.02 a
Net reproductive rate (*R*_0_) (offspring)	1.13 ± 0.39 b	17.63 ± 4.49 a	10.23 ± 2.83 a
Mean generation time (*T*) (d)	23.79 ± 3.31 a	20.89 ± 0.79 a	19.60 ± 0.33 a
Gross reproductive rate (GRR)	6.18 ± 1.51 b	90.32 ± 16.80 a	30.76 ± 6.63 ab

**Note:**

Standard errors were estimated by using 200,000 bootstrap replicates. Means marked with different letters are significantly different between three treatments using the paired bootstrap test at the 5% significant level.

### Survival rate

**Figure 2:**
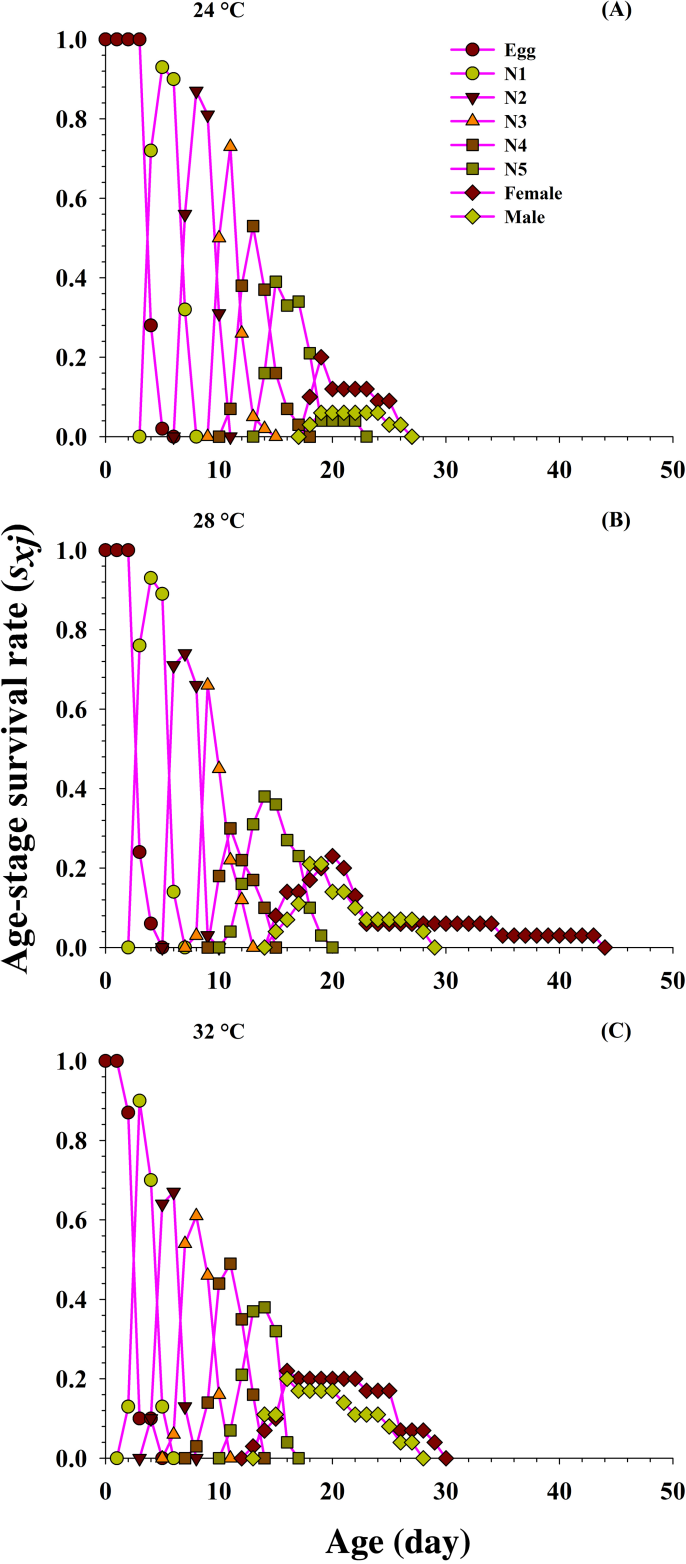
Influence of three different temperatures ((A) 24 °C, (B) 28 °C and (C) 32 °C) on the age-stage-specific survival rate (*s*_xj_) of the *O. strigicollis*. *N*1 = First Instar, *N*2 = Second Instar, *N*3 = Third Instar, *N*4 = Fourth Instar and *N*5 = Fifth Instar.

From the detailed age-stage survival rate (*S_xj_*) of *O. strigicollis* fed on the *P. gossypiella* at different temperatures (Fig. 2), our results indicate the probability of a newly hatched predator living to age *x* and stage *j*. Significant difference was also observed in overlapping plotted curves for different developmental stages because developmental rate varied among individuals at different temperatures. The projected curves revealed entirely diverse patterns for each developmental stage at each temperature. As the age increased, the survival rate of individuals gradually decreased and showed an inverse relation at each temperature. The developmental time of *O. strigicollis* male and female was longer and survival rate was lower at 24 °C, compared with other two temperatures (28 °C and 32 °C). The survival peak was higher for male (18 d, 0.21) and female (20 d, 0.23) at 28 °C and 32 °C (male; 16 d, 0.20 and female; 16 d, 0.22) than at 24 °C (male; 19 d, 0.06 and female; 19 d, 0.20) when fed on *P. gossypiella* eggs.

**Figure 3:**
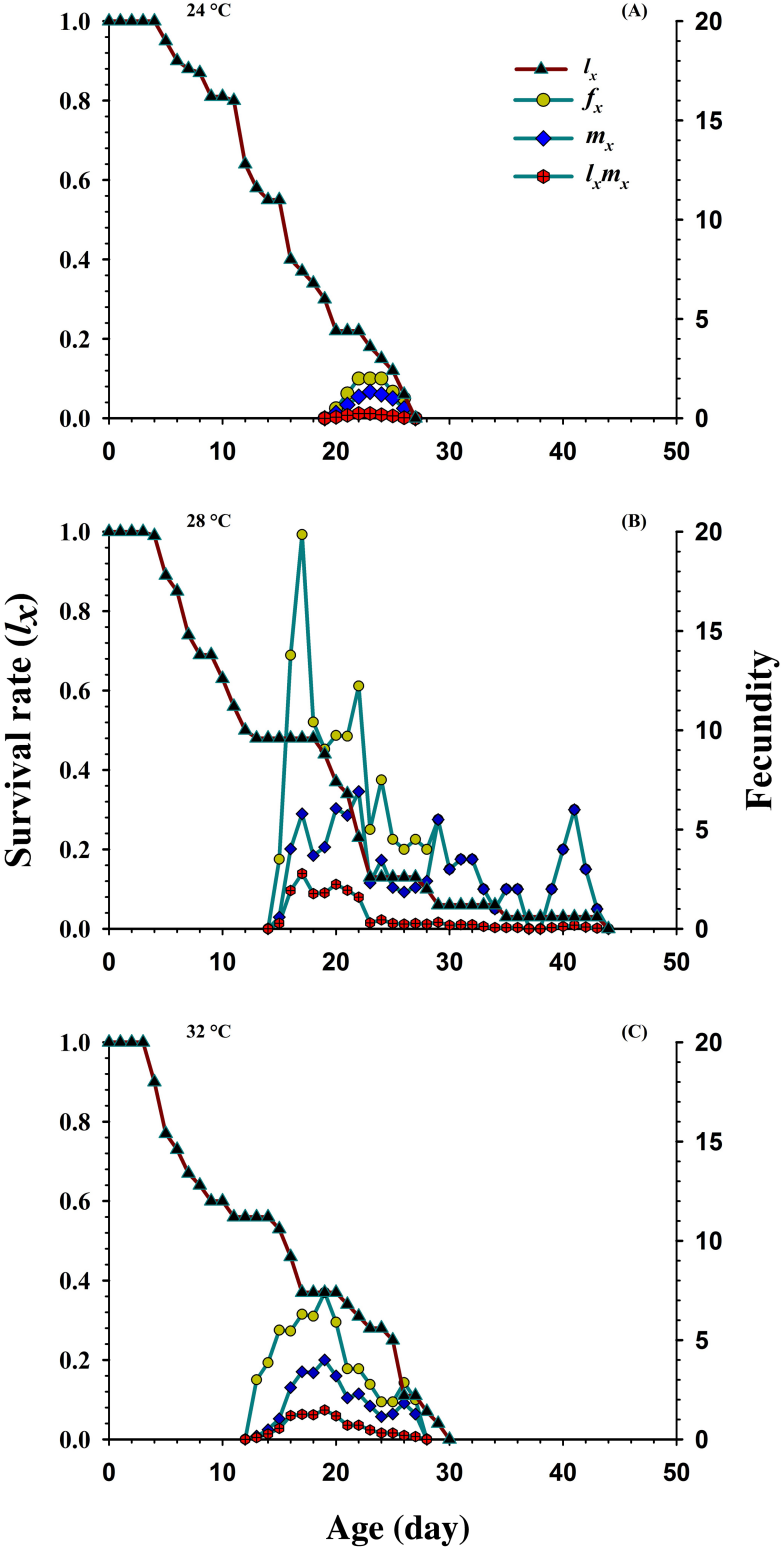
Influence of three different temperatures ((A) 24 °C, (B) 28 °C and (C) 32 °C) on the age-specific survival rate (*l*_x_), female age-specific fecundity (*f*_x_), age-specific fecundity (*m*_x_) and age specific maternity.

Figure 3 shows the age-specific survival rate (*l_x_*), the age-specific fecundity of total population (*m_x_*), age-stage specific fecundity (*f_xj_*) and the age-specific maternity (*l_x_m_x_*) curves plotted at different temperatures. The curves of *l_x_* (basic form of the age-stage survival rate *S_xj_*) declined or showed inverse relation at 24 °C and 32 °C, but it was directly proportional to 28 °C. The peak recorded values of age-stage specific fecundity (*f_xj_*) was (22 d, two eggs), (17 d, 19.85 eggs) and (19 d, 7.4 eggs) appeared at 24, 28 and 32 °C, respectively. The curves of *m_x_* showed that reproduction began relatively earlier at 32 °C (age; 12 d), than at 28 °C (age; 14 d) and 24 °C (age; 19 d). But, the highest recorded *m_x_* peak was (seven eggs per individual; age 22 d) at 28 °C than 24 °C (1.33 eggs per individual; age 23 d) and 32 °C (4.00 eggs per individual; age 19 d).

### Life expectancy

**Figure 4:**
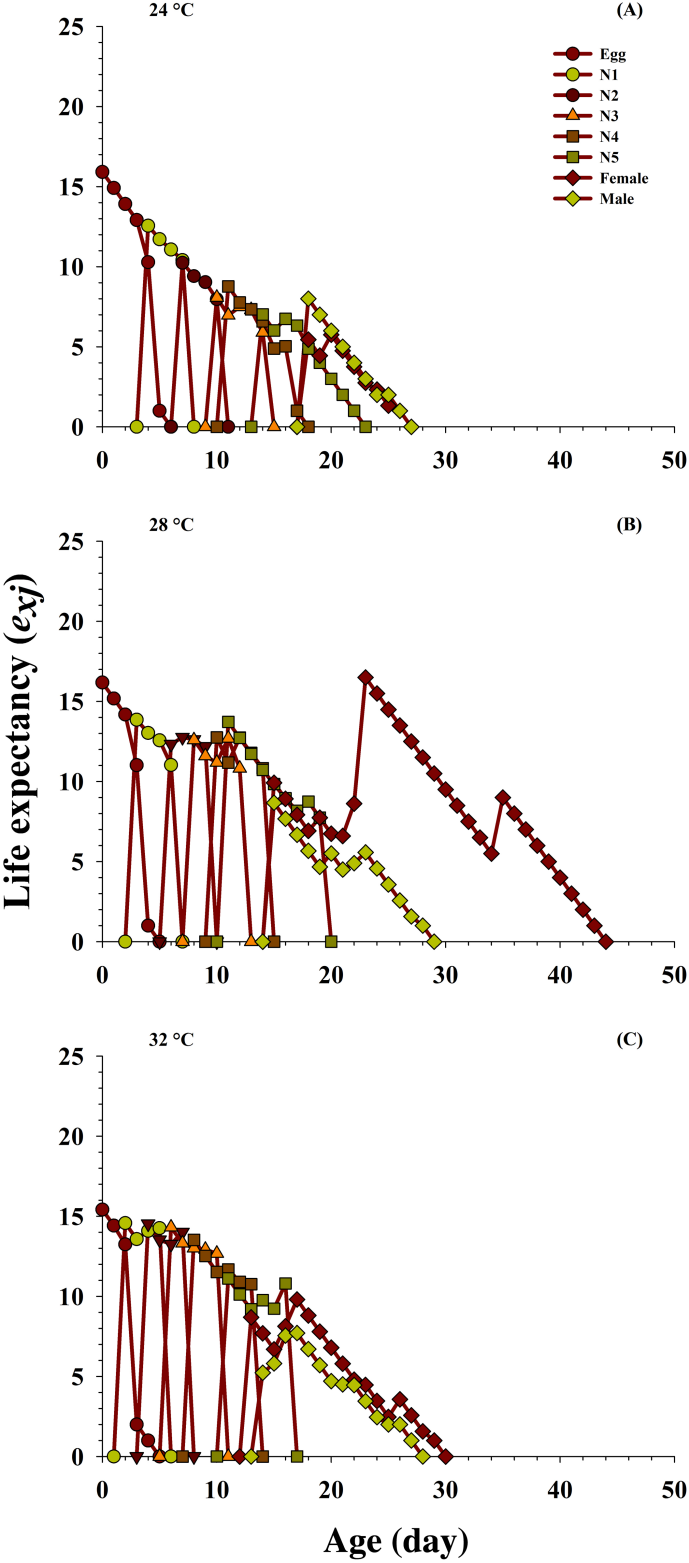
Influence of three different temperatures ((A) 24 °C, (B) 28 °C and (C) 32 °C) on the age-stage-specific life expectancy (*e*_xj_) of the *O. strigicollis*. *N*1 = First Instar, *N*2 = Second Instar, *N*3 = Third Instar, *N*4 = Fourth Instar and *N*5 = Fifth Instar.

The effects of the different temperatures on the predicted average life expectancy (*e_xj_*) of the population at every stage of *O. strigicollis* (Fig. 4) were determined. The longevity of the newly hatched eggs of *O. strigicollis* at age zero was 15.92 d at 24 °C, 16.18 d at 28 °C and 15.41 d at 32 °C when fed on *P. gossypiella* eggs. With variation in other developmental stages, an increasing trend in the female adult expectation was observed (16.5 d, age 23 days) at 28 °C than those at the other two temperatures: 24 °C (5.75 d, age 20 days) and 32 °C (9.80 d, age 17 days). Overall, the highest life expectancy was recorded at 28 °C compared with those at 24 °C and 32 °C.

### Reproductive value

The age-stage reproductive value (*V_xj_*) describes the part of an individual of age *x* and stage *j* toward the upcoming population (i.e., the scale of population forecasting). The results reveal that the curves for *V_xj_* significantly increased (8.48 d^−1^ on 20 d to 51.68 d^−1^ on 15 d) at 24 °C and 28 °C, respectively, but decreased (22.65 d^−1^ on 13 d) at 32 °C when female emerged.

**Figure 5:**
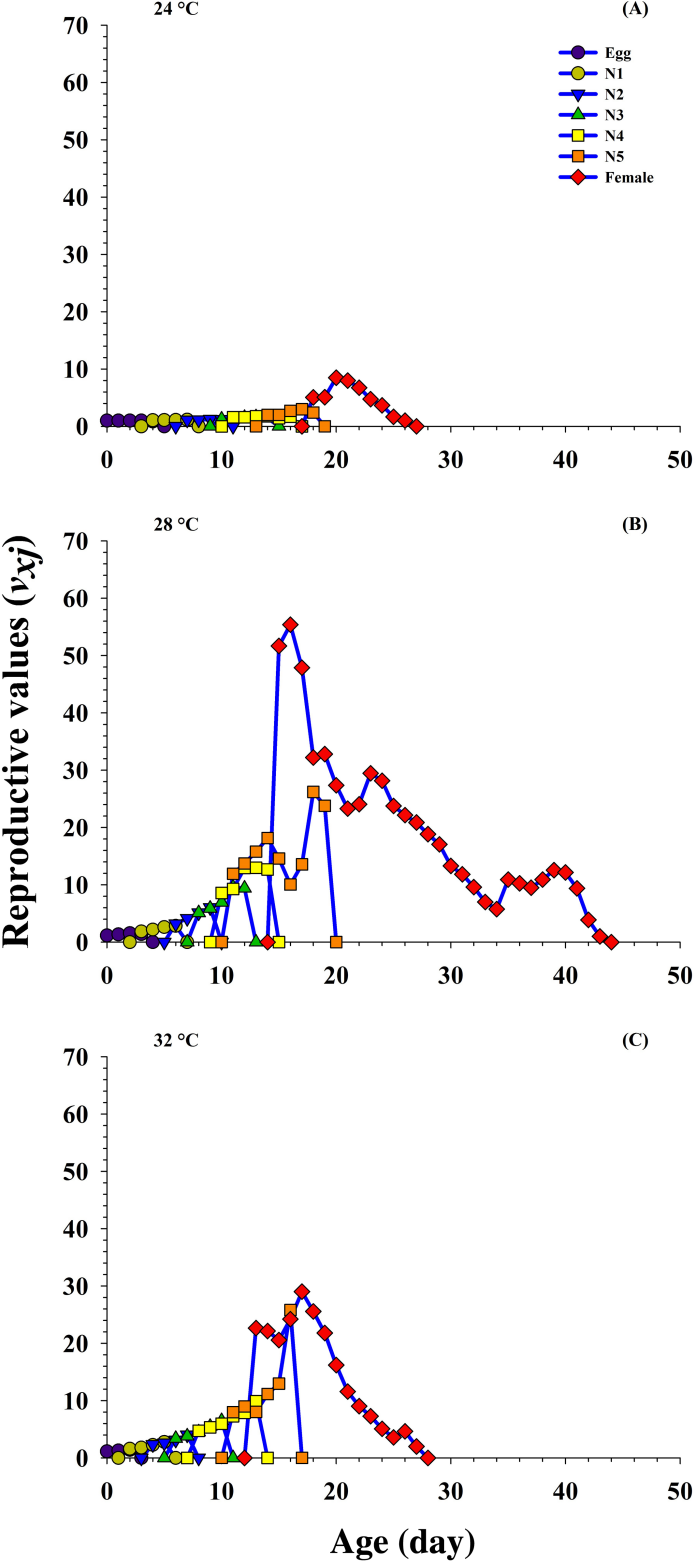
Influence of three different temperatures ((A) 24 °C, (B) 28 °C and (C) 32 °C) on the age-stage reproductive value (*v*_xj_) of the *O. strigicollis*. *N*1 = First Instar, *N*2 = Second Instar, *N*3 = Third Instar, *N*4 = Fourth Instar and *N*5 = Fifth Instar.

In addition, the *V_xj_* is exactly the same as the finite rate, that is, 1.00 d^−1^ at 24 °C, 1.15 d^−1^ at 28 °C and 1.13 d^−1^ at 32 °C when fed on *P. gossypiella* eggs. The results reveal that the *V_xj_* was higher at 28 °C, and showed that the *P. gossypiella* had a more positive effect on *O. strigicollis* reproduction than 24 °C and 32 °C (Fig. 5).

### Prey preference

**Figure 6:**
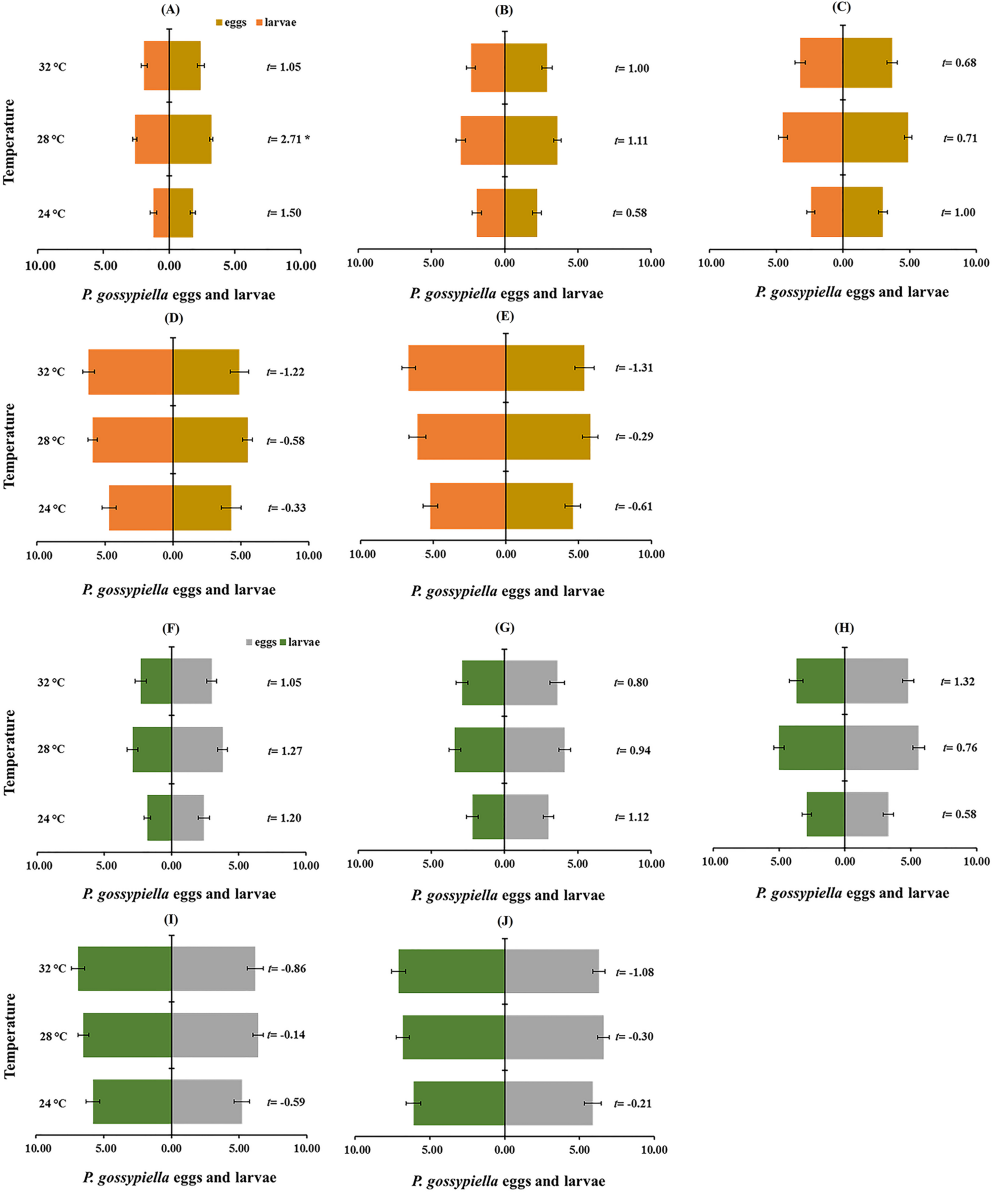
Prey preference between *P. gossypiella* eggs and first instar larvae (10 each) of the predatory stages of *O. strigicollis* at different temperatures (24 °C, 28 °C and 32 °C) after 12 h ((A) Third instar, (B) fourth instar, (C) fifth instar, (D) male and (E) female) and 24 h interval ((F) Third instar, (G) fourth instar, (H) fifth instar, (I) male and (J) female); using paired *t*-test, *n* = 10, *P < 0.05, **P < 0.01.

The prey preference among *P. gossypiella* eggs and first instar larvae was evaluated by paired *t*-test (Figs. 6A and 6B). Male and female preferences were not different, and they preferred first instar larvae of *P. gossypiella* than other predatory stages. The other predatory stages (third, fourth and fifth instars) preferred *P. gossypiella* eggs rather than first instar larvae after 12 h (Fig. 6A) and 24 h (Fig. 6B) at each temperature.

## Discussion

The two-sex life table is the best tool/technique for basic and vital research in ecological studies compared with traditional life tables (Jaleel et al., 2018a, 2018b; Ali et al., 2020). To our knowledge, the present study for the first time describes the feeding potential and two-sex life table traits of *O. strigicollis* fed on *P. gossypiella* eggs at different temperatures. The results of the present study will help to boost IPM strategies against *P. gossypiella* by providing more detailed basic and additional information, such as temperature preference and fitness of this predator.

Insects are sensitive to temperature fluctuations, and temperature can have adverse effects on feeding, development, biology, physiology, and behavior of insects, which in turn affect insect fitness (Barbosa et al., 2019; Hafeez et al., 2019a). The feeding potential of predators in different ecological and biological systems is considered a basic element reflecting their feeding or killing efficiency on particular prey or hosts. This feature particularly encourages or supports the predator–prey relationship to check the fitness on the basis of food preference (best or poor-quality food) on different preys under different environmental conditions (Zappalà et al., 2013; Brown & Mathews, 2014). The feeding potential of different *Orius* species on different prey species has been recorded in many previous studies (Kakimoto et al., 2005; Aragón-Sánchez et al., 2018; Ali et al., 2020). In the present study, the feeding potential of *O. strigicollis* adult stages (males and females) showed greater potential when fed on *P. gossypiella* eggs compared with those fed on first instar larvae of *P. gossypiella*. Our results agree with those of a previous study (Aragón-Sánchez et al., 2018), in which the authors explained that *O. laevigatus* adult stages consumed more *Spodoptera exigua* eggs than larvae. Previous studies have reported the effects of different temperatures on the survival and development of various insects (Zhang et al., 2012; Garrad, Booth & Furlong, 2016). Significant distinctions in the nymphal development of each instar showed that all nymphal stages of *O. strigicollis* were susceptible to changing the constant temperature regime. Many studies reported that the nymphal developmental duration of *Orius* spp. decreased when temperature increased (Cocuzza et al., 1997; Zhou et al., 2006).

In the present study, the minimum total nymphal duration was observed when the temperature increased (Table 2). The conflicting relationship between longevity and temperature was observed in *Orius* species. Kakimoto et al. (2005) revealed that the longevity of *O. strigicollis* females was longer at 23 °C (73.1 days) compared with that at 29 °C (27.2 days). In another study, Zhang et al. (2012) demonstrated that longevity decreased with increasing temperature from 25 °C to 31 °C when the adults of *O. similis* were fed on *T. cinnabarinus*. The same trend was observed in the present study with little contrast, where the total longevity of the female and male adults of *O. strigicollis* decreased and changed according to temperature, except at 28 °C where the total longevity of females was higher (26.12 days) and there was no significant difference between the three treatment temperatures. This result may be attributed to the nutritional content of particular diets/hosts, increased feeding, different temperatures (suitable for females) to egg laying and to complete metabolic reactions in the female body (Saeed et al., 2019; Ali et al., 2020). We recorded that the females lived longer than the males, and the total longevity of males at 24, 28 and 32 °C was 26, 23.67 and 21.25 d, respectively, whereas the total longevity of females at 24, 28 and 32 °C was 23.83, 26.12 and 23.33 d, respectively, which agreed with the findings of previous studies (Zhou et al., 2006; Zhang et al., 2012).

In the present study, the pre-oviposition period (APOP) of *O. strigicollis* was found to be temperature dependent. This is a promising outcome that agrees with a previous study (Zhang et al., 2012), in which it was reported that the pre-oviposition period of *O. similis* fed on *T. cinnabarinus* decreased when temperature increased from 25 °C to 31 °C. However, in our study, the length of APOP significantly decreased, which showed that *O. strigicollis* is capable of rapidly completing the development of the reproductive system (ovarioles) compared with those at low temperatures when *P. gossypiella* eggs were provided as food. The maximum mean daily oviposition of *O. similis* was recorded at 30 °C compared with 18 °C when fed on aphid species as prey documented by Ahmadi, Sengonca & Blaeser (2007). Similarly, Sengonca, Ahmadi & Blaeser (2008) reported the maximum mean daily oviposition of *O. similis* when fed on *M. persicae* (5.6 eggs/day) than on *A. gossypii* (2.9 eggs/day) at 25 °C. These results show that the food also influenced oviposition in insects, which agreed with the results of our study in which the oviposition period differed according to diet and temperature. An early oviposition period at high temperature is possibly the expression of an increased metabolic rate (Barbosa et al., 2019; Jaleel et al., 2020). Fecundity normally plays significant roles in insect population dynamics (Jaleel, Lu & He, 2018). The variation trend in fecundity was similar to the oviposition period of female *O. strigicollis* and was mainly associated with temperature in the present study. The highest lifetime fecundity (overall oviposition per female) was observed at 28 °C in our study. This result shows the potential of *O. strigicollis* optimum temperature for egg laying, which agrees with the results of a previous study (Kakimoto et al., 2005), in which three different *Orius* species were fed on *Ephestia kuehniella* at 17, 20, 23, 26 and 29 °C, and among these tested temperatures, the highest fecundity was observed at the highest temperature (Table 2).

For growth, development, and survival of an insect, *r* is an important and critical demographic parameter (Varley & Gradwell, 1970; Chen et al., 2017). The *r* is greatly linked with the vulnerability of a host, prey, or diet to insect feeding (Musa & Ren, 2005). According to demographic life table theory, if *r* is greater than zero (0), then the prey (host) is suitable for population growth (Southwood & Henderson, 2009; Chen et al., 2017). This theory supported the results of the present study, and surprisingly showed a higher *r* for *O. strigicollis* (faster development and higher survival rates) at 28 °C owing to higher fecundity and shorter development time than other treated groups (24 °C and 32 °C) (Table 3). In addition, our results agreed with those of another study in which the recorded values of *r* were 0.08, 0.10 and 0.12 per day at 25, 28 and 31 °C, respectively, when *O. similis* was fed on *T. cinnabarinus* (Zhang et al., 2012). Similarly, Kakimoto et al. (2005) reported the *r* for three different *Orius* species and indicated that temperature and diet play significant roles in the population parameters of insects.

The *R*_0_ is also a significant indicator of population development where the highest rate of population is dependent upon and directly related to the number of eggs (Sayyed et al., 2008). The *GRR* is considered a sign or concept of a rapid increase in insect population that depends on fecundity and adult eclosion. Generally, these parameters are affected by food source and temperature (Huang & Chi, 2013; Chen et al., 2018a). In the present study, the highest net reproductive rates (*R*_0_) and *GRR* were achieved when bugs were fed on *P. gossypiella* eggs at 28 °C (Table 3). Such high growth rates must be due to the rapid development and high fecundity of *O. strigicollis*. In addition, our results were somehow in contrast to those of some previous studies that recorded different *Orius* species at different temperatures and diets (Kakimoto et al., 2005; Hamdan, 2012; Ali et al., 2020), which was attributed to the temperature that ultimately and directly affects the parameters of insect populations. The temperature affected the biotic potential of *O. strigicollis* as shown by the *R*_0_, *r*, and *GRR* at 28 °C (Table 3). The population increased only when *R*_0_ was greater than one (Southwood & Henderson, 2009; Chen et al., 2017), and our results were also according to this theory.

## Conclusion

In our study, we presented the feeding potential of *O. strigicollis* on *P. gossypiella* eggs and first instar larvae and the fitness traits of *O. strigicollis* when fed on *P. gossypiella* eggs (diet) at three different temperatures by age-stage, two-sex life tables, which could provide more useful insights for mass rearing these bugs. The present study will support future studies on the biocontrol or environmentally friendly control of *P. gossypiella* in IPM programs. We found that temperature has multi-dimensional effects on the reproductive capacity and development potential of *O. strigicollis*, which showed the feeding potential, maximum growth, survival and fecundity at 28 °C compared with those at the other tested temperatures. Therefore, a plan for the IPM of noctuid moth could be conducted in a timely manner, and this information can be used to assess the suitability of *O. strigicollis* as a natural enemy under different environmental conditions. The present study would be helpful to future work aiming to determine the optimum way to rear these bugs for efficient control of pests according to the different environmental conditions in greenhouse and field conditions.

## Supplemental Information

10.7717/peerj.9594/supp-1 Supplemental Information 1 Two-Sex life table data sheet at 24 °C, 28 °C and 32 °C.

10.7717/peerj.9594/supp-2 Supplemental Information 2 Feeding potential on *P. gossypiella* eggs after 24 h, larvae after 12h and 24 h data sheet.

10.7717/peerj.9594/supp-3 Supplemental Information 3 24, 28 and 32 C temp prey preference 12 and 24 h data sheet.

10.7717/peerj.9594/supp-4 Supplemental Information 4 Highlights of study.

## Additional Information and Declarations

### Data Availability

The following information was supplied regarding data availability: The raw data is available in the Supplemental Files.
