# Supplementary material for: Determination of fitness traits of Orius strigicollis Poppius (Hemiptera: Anthocoridae) on Pectinophora gossypiella (Lepidoptera: Gelechiidae) using two-sex life table analysis
Source: PeerJ. 2020 Aug 17;8:e9594. doi: 10.7717/peerj.9594 (PMC7439961; doi:10.7717/peerj.9594)
Supplement: Supplemental Information 4 [file peerj-08-9594-s004.docx]

**Highlights**

- Study focus the feeding potential of *O. strigicollis* predatory stages (i.e., third instar, fourth instar, fifth instar, male and female) on *P. gossypiella* eggs and first instar larvae.
- Study focus the fitness traits of *O. strigicollis* feeding on *P. gossypiella* eggs at three temperatures (24, 28 and 32°C) in laboratory that have not been tested before.
- Study focus the prey preference of *O. strigicollis* predatory stages (i.e., third instar, fourth instar, fifth instar, male and female) on *P. gossypiella* eggs and first instar larvae.
- The value of population parameters i.e., intrinsic rate of increase (*r*) and the net reproductive rate (*R_0_*) was higher at 28°C that shows the more potential to survive and reproductive capability of bugs.
- The *P. gossypiella* eggs were more preferred stages than first instar larvae.
